# Ordered chromatin changes and human X chromosome reactivation by cell fusion-mediated pluripotent reprogramming

**DOI:** 10.1038/ncomms12354

**Published:** 2016-08-10

**Authors:** Irene Cantone, Hakan Bagci, Dirk Dormann, Gopuraja Dharmalingam, Tatyana Nesterova, Neil Brockdorff, Claire Rougeulle, Celine Vallot, Edith Heard, Ronan Chaligne, Matthias Merkenschlager, Amanda G. Fisher

**Affiliations:** 1 Lymphocyte Development Group, MRC Clinical Sciences Centre, Imperial College School of Medicine, Hammersmith Hospital Campus, Du Cane Road, London W12 0NN, UK; 2 Microscopy Laboratory, MRC Clinical Sciences Centre, Imperial College School of Medicine, Hammersmith Hospital Campus, Du Cane Road, London W12 0NN, UK; 3 Bioinformatics and Computing facility, MRC Clinical Sciences Centre, Imperial College, London W12 0NN, UK; 4 Developmental Epigenetics Group, Department of Biochemistry, University of Oxford, South Parks Road, Oxford OX1 3QU, UK; 5 Epigenetics and Cell Fate, CNRS UMR7216, Université Paris Diderot, Sorbonne Paris Cité, 75013 Paris, France; 6 Mammalian Developmental Epigenetics Group, Institut Curie, 26 rue d'Ulm, 75248 Paris, France

## Abstract

Erasure of epigenetic memory is required to convert somatic cells towards pluripotency. Reactivation of the inactive X chromosome (Xi) has been used to model epigenetic reprogramming in mouse, but human studies are hampered by Xi epigenetic instability and difficulties in tracking partially reprogrammed iPSCs. Here we use cell fusion to examine the earliest events in the reprogramming-induced Xi reactivation of human female fibroblasts. We show that a rapid and widespread loss of Xi-associated H3K27me3 and *XIST* occurs in fused cells and precedes the bi-allelic expression of selected Xi-genes by many heterokaryons (30–50%). After cell division, RNA-FISH and RNA-seq analyses confirm that Xi reactivation remains partial and that induction of human pluripotency-specific *XACT* transcripts is rare (1%). These data effectively separate pre- and post-mitotic events in reprogramming-induced Xi reactivation and reveal a complex hierarchy of epigenetic changes that are required to reactivate the genes on the human Xi chromosome.

## Abstract (web summary)

Reactivation of the inactive X chromosome (Xi) has modelled epigenetic reprogramming in mouse. Here, by using cell fusion between human female fibroblasts and mouse embryonic stem cells, the authors show a complex hierarchy of epigenetic changes that are required to reactivate the genes on the human Xi chromosome.

## Graphical abstract

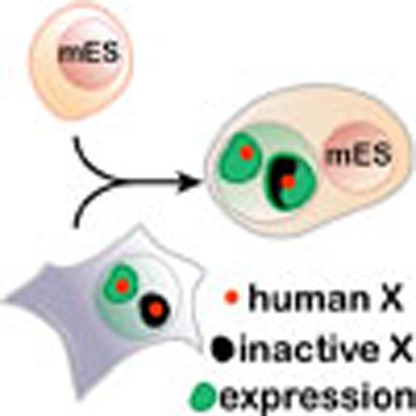

X chromosome inactivation (XCI) is an exemplar of epigenetically regulated silencing used by mammals to compensate for gene dosage between males (XY) and females (XX)^1^. XCI is a multi-factorial and multi-step process that is established as pluripotent cells of the embryo differentiate and can be reversed when somatic cells re-acquire pluripotency^2^. Non-coding *Xist* RNA initiates inactivation by coating the presumptive inactive X chromosome (Xi)^3^ and creating a nuclear domain from which RNA polymerase II and activating chromatin marks are excluded^4^,^5^. Several repressive histone and DNA modifications including histone H3 lysine 27 tri-methylation (H3K27me3) and 5-methyl-cytosine are incorporated within the chromatin of the Xi and result in the stabilization of gene silencing^6^,^7^,^8^. Prior studies that have examined the contribution of different XCI factors to this silencing have shown that removal of *XIST*/*Xist*^9^,^10^ or H3K27me3 (refs 11, 12) in somatic cells is insufficient to reverse XCI but can lead to sporadic gene reactivation when coupled to experimental perturbation of histone acetylation or DNA methylation^13^. Collectively such experiments contribute to the classical view that components such as *XIST/Xist* and polycomb-mediated histone modifications are required for the initiation but not the maintenance of Xi silencing. Recently, however, it was shown that loss of *Xist*, when combined with multiple cell divisions, could lead to the consistent reactivation of some genes along the Xi chromosome^14^. This suggests that while control of Xi silencing is multi-layered and partially redundant, different domains on the Xi may vary in susceptibility to epigenetic reactivation.

In somatic cells the induction of pluripotency can result in reactivation of the silent Xi. A strong correlation between pluripotency and X chromosome reactivation (XCR) has been shown in mouse in experimental reprogramming studies, as well as *in vivo* during normal pre-implantation and primordial germ cell development^15^. Model systems where XCI and its reversal can be induced have allowed the molecular interactions between the pluripotency network and XCI to be dissected. For example, several mouse pluripotency-associated transcription factors including Oct4, Nanog and Rex1 have been shown to control XCI by regulating the transcription of *Xist* or its antagonist *Tsix*^2^,^16^,^17^,^18^. In addition, a better understanding of the contribution of different factors in maintaining Xi gene silencing has been achieved by identifying the intermediate steps and the dynamics of XCR during somatic cell reprogramming^19^. Comparable studies in human cells have been more problematic. This arises from the fact that human embryonic stem cells (hESCs) and reprogrammed human induced pluripotent stem cells (hiPSCs) with two active X chromosomes are notoriously unstable in culture^20^,^21^,^22^,^23^,^24^. Cultured human pluripotent cells are prone to undergo unscheduled re-initiation of XCI^25^,^26^, followed by epigenetic ‘erosion' and inappropriate loss of silencing^27^. Dynamic studies of XCR in human cells have, therefore, been hampered by epigenetic instability in culture and the consequent selective pressures that environmental conditions, such as oxygen tension, impose (reviewed in ref. 28). To circumvent these limitations, we have utilized cell–cell fusions of somatic and ESCs to generate transient human–mouse heterokaryons in which the earliest steps of pluripotency-induced reprogramming could be followed.

Previously, studies of fusions between cells of different types had provided some of the first evidence that XCR was associated with pluripotency^29^. In early studies, in which human somatic and rodent pluripotent and embryonic carcinoma cells were fused to generate stable hybrid cell clones, reactivation of human Xi-linked genes was demonstrated and enhanced by treatment with drugs that inhibited DNA methylation^30^,^31^,^32^,^33^. These studies also noted that in several human–rodent hybrid lines the distribution of *XIST* RNA was also perturbed^3^,^34^,^35^, raising concerns that there might be an intrinsic incompatibility between human and mouse/hamster/rat cells. Here we used cell fusion to examine the earliest events in human XCR. Rather than examining conventional human–rodent hybrids that contain a limited human chromosome contribution^36^, we analysed XCI in newly formed heterokaryons before and just after the first mitosis. This system has the advantages of being able to monitor the immediate events in reprogramming with higher efficiencies than the iPSC system and to track cells undergoing reprogramming more easily.

To investigate the dynamics and extent of human XCR induced by pluripotent reprogramming we fused human female fibroblasts (hF) with mouse embryonic stem cells (mESCs). Previous studies have shown that following cell fusion an ensemble of mESC-factors is available to the human nucleus and that this stimulates a rapid reactivation of the human pluripotency network, accompanied by global chromatin changes and the functional resetting of lineage potential^37^,^38^. Here we show that immediately after cell fusion, human and mouse genomes remain separate until the first mitosis when hybrid cells arise^39^, and that human pluripotency genes are re-expressed ahead of cell division. During this early period, we show that human nuclei undergo a progressive loss of H3K27me3 and *XIST* from the Xi and selectively re-express certain human Xi genes. These data suggest that *XIST* loss although necessary may be insufficient for Xi reactivation, and reveal that that reprogramming of human female somatic cells can induce the reactivation of specific Xi genes ahead of mitosis.

## Results

### Pluripotent reprogramming of human female fibroblasts

**Figure 1:**
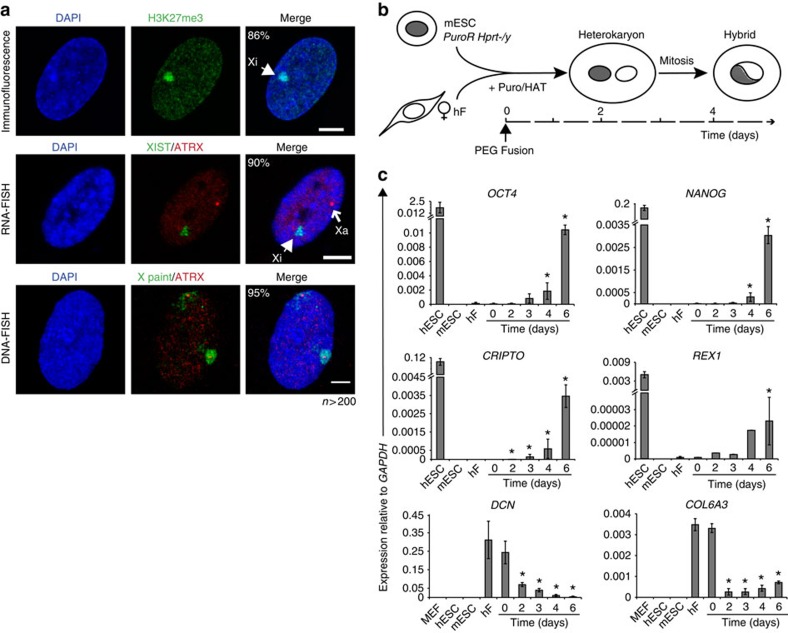
Human female XaXi fibroblasts are reprogrammed via cell-fusion with mouse embryonic stem cells. (**a**) Confocal images of normal XaXi female hF (IMR90) where the Xi is visible as a DAPI-dense region, enriched for H3K27me3 histones (arrowhead, top panels) and coated by *XIST* RNA (arrowhead, middle panels). The Xa is instead revealed by *ATRX* nascent transcript detection (open arrow, middle panels). Two X chromosomes were confirmed by labelling the *ATRX* locus and the whole X chromosome using DNA-FISH (bottom panels; ≥95% in primary or *TERT*-overexpressing cells). Total number of cells (*n*) was >200 per experiment in at least two independent cultures. Scale bar, 5 μm. (**b**) Scheme representing the strategy for reprogramming human fibroblasts towards pluripotency. hF are fused with a male mESC line (E14Tg2a) that is sensitive to HAT (*Hprt*^*−/y*^) and resistant to puromycin (*PuroR*). Heterokaryons containing discrete nuclei from both partners were selected by HAT/puromycin and generate drug-resistant hybrids after the first cell division. (**c**) Histogram plots showing the expression of human pluripotency genes (*OCT4*, *NANOG*, *CRIPTO* and *REX1*) and fibroblast-specific genes (*DCN*, *COL6A3*) in the HAT/puromycin resistant cultures where unfused hF, mESCs, H1 hESCs and primary mouse embryonic fibroblasts (MEF) provide controls. Gene expression is reported as 2^−ΔCt^ relative to *GAPDH* and represents the average of 3–10 independent experiments. Error bars indicate s.e.m. and (*) mark values that significantly differ from hF/day 0 (*P*≤0.05, two-sided *t*-test). See also Supplementary Fig. 1.

In order to investigate human XCR during pluripotent reprogramming we first examined the epigenetic signatures of the two X chromosomes in female diploid fibroblasts by fluorescence *in situ* hybridization (FISH), 4,6-diamidino-2-phenylindole (DAPI) staining and the distribution of modified histones (Fig. 1a). In the nuclei of female hF, the Xi is condensed during interphase and forms a heterochromatin compartment identified as the DAPI-dense Barr body. This compartment is coated by *XIST* RNA and enriched in H3K27me3, as well as other histone modifications associated with silencing^6^. Before reprogramming, karyotype analysis and DNA-FISH revealed that most fibroblasts had two X chromosomes (>90%) and labelling with anti-H3K27me3 antibodies clearly identified a single Barr body in 86% of cells (Fig. 1a). Further confirmation was provided by simultaneous RNA-FISH labelling with probes recognizing *XIST* and *ATRX* (an X-linked gene), where *XIST* RNA painted the Xi and nascent *ATRX* transcripts marked the location of the active X chromosome (Xa) (Fig. 1a, middle panels). Fibroblasts were then immortalized by human *TERT* transduction to alleviate senescence^24^ and the Xi status was revalidated before reprogramming.

Female hF and male mESCs were fused in equal ratio and the kinetics of reprogramming and XCR was followed in heterokaryon and hybrid cells generated over the next 6 days. To identify and enrich for fused (hFxmESC) cells we used two different approaches. In the first approach, *Hprt*-null mESCs that carry a puromycin resistance gene (*PuroR*) were fused with primary or *TERT*-immortalized human fibroblasts and inter-species heterokaryons were selected by HAT/puromycin (Fig. 1b). Alternatively, we directly isolated fused cells based on the simultaneous expression of histone H2B-mCherry (mESC) and HP1α-green fluorescent protein (hF), as shown in Supplementary Fig. 1a. In both reprogramming models, we confirmed an up-regulation of human pluripotency genes (*OCT4*, *NANOG*, *CRIPTO*, *REX1*) and a down-regulation of human fibroblast-associated genes (*DCN*, *COL6A3*) (Fig. 1c and Supplementary Fig. 1b), as previously reported^37^,^40^,^41^. Analysis of fused hFxmESC (H2B-mCherry+/HP1α-green fluorescent protein (GFP)+ cells, enriched by fluorescence-activated cell sorting) showed that human pluripotency gene expression was evident 2 days after fusion at a time when there is no cell division (Supplementary Fig. 1c). Live-cell imaging of these cultures confirmed that fusion between individual nuclei within heterokaryons (green–red) generates hybrids from day 3 onwards as some fused cells begin to enter their first mitosis (Supplementary Fig. 1d,c, hybrid cells increase from <5% at day 2 to 45% at day 4). This approach, therefore, allowed us to examine the earliest events in reprogramming-mediated Xi reactivation that occurred ahead of or immediately following the first cell cycle.

### Reprogramming triggers global changes in human Xi chromatin

**Figure 2:**
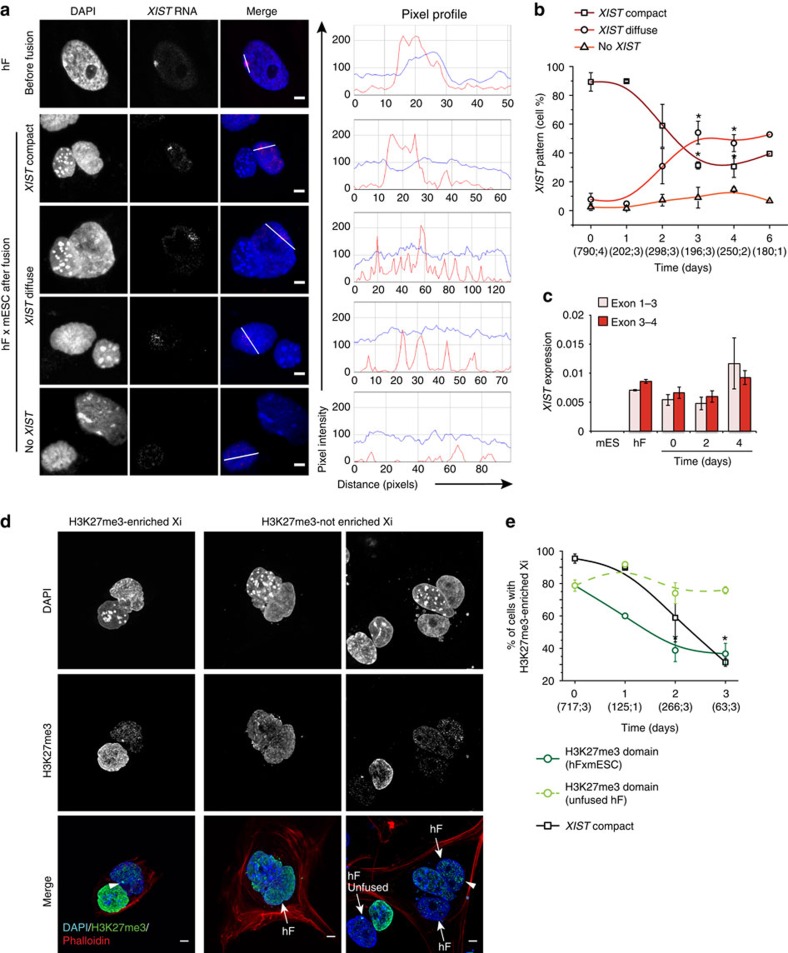
*XIST* RNA delocalization and loss of H3K27me3 enrichment at the Xi are early events in cell fusion-mediated reprogramming. (**a**) Confocal images showing *XIST* RNA distribution in hF nuclei before and after fusion with mESCs. Three distinct *XIST* patterns were discriminated based on the pixel profile of the signal: a ‘compact' *XIST* signal spanning at least 2 μm with constant pixel intensity; a ‘diffuse' signal with discontinuous peaks (< 2 μm); and a signal with same intensity as background, classified as ‘no *XIST*'. Profiles show the pixel intensity for DAPI staining (blue) and *XIST* signal (red) along the white line traced through the nucleus. Image scale=6,247 pixels μm^−1^. (**b**) Percentage of human nuclei showing the three different *XIST* patterns represented in (**a**) at successive days after mESC-fusion. At day 3, fused cells were exclusively heterokaryons (homokaryons and unfused cells not scored). From day 4–6, hybrid cells were detected at increasing frequency (60–92%). Data-points represent the average of independent biological replicates±s.e.m. and curves show B-spline fitting. (*) indicates significant changes versus day 0 (*P*≤0.05; two-sided *t*-test). Total number of scored cells and number of replicate experiments are in parenthesis along the *x* axis. (**c**) Abundance of *XIST* spliced RNA measured by RT–qPCR with two different human-specific primer sets that span distinct exon–exon junctions. Data are reported as 2^−ΔCt^ relative to *GAPDH* and represent the mean of three independent experiments±s.e.m. No significant difference was detected (*P*>0.05; two-sided *t*-test) (**d**) Confocal images of hFxmESC heterokaryons at day 3 with (arrowhead) and without a H3K27me3-enriched nuclear domain. Scale bar, 5 μm. (**e**) Percentage of hFxmESC heterokaryons (dark green circles) or unfused hF on the same slide (light green circles) with a H3K27me3-enriched nuclear domain at 0, 1, 2 and 3 days after fusion. Data are represented as in (**b**) and percentage of heterokaryons with a compact *XIST* signal (black squares) are shown within the plot to facilitate comparison. See also Supplementary Fig. 2.

As *XIST* RNA is a key regulator of XCI we analysed its distribution in human nuclei before and after fusion with mESCs (Fig. 2a). Before fusion, most human fibroblasts showed a compact *XIST* signal (Fig. 2a) that overlapped the X chromosome territory (Supplementary Fig. 2a). After fusion, an increasing proportion of cells showed a diffuse *XIST* RNA signal that was no longer confined to the territory of the human X chromosome (Supplementary Fig. 2b). By day 3, 50% of heterokaryons had diffuse *XIST* signals (Fig. 2a,b and Supplementary Fig. 2b) and a minority of cells (≈10%) had no detectable *XIST*. The proportion of fused cells showing diffuse *XIST* signals remained reasonably constant over the next few days (day 4–6) as the number of hybrid cells increased. As *Xist* expression has been shown to diminish during mouse iPSC reprogramming^42^, we assessed the level of the spliced human *XIST* transcripts by quantitative reverse transcription (qRT)–PCR. *XIST* transcript levels did not appreciably change over 4 days following cell fusion (Fig. 2c), suggesting that *XIST* RNA is delocalized, but is not silenced during reprogramming. To verify that *XIST* delocalization was a specific response to pluripotent conversion rather than a response to heterokaryon formation *per se*, we fused female hF with mouse B (mB) cells and assessed human *XIST* distribution upon culture in mouse ESC medium. In these fused cells, human *XIST* RNA remained tightly localized with the DAPI-dense presumptive Xi in the majority of heterokaryons analysed at day 3 (Supplementary Fig. 2c, quantified in Supplementary Fig. 2d). At later time points (day 6) we observed a decline in hFxmB cells showing compact XIST signals (Supplementary Fig. 2d, grey) consistent with cells entering mitosis and loosing chromosome-bound *XIST* RNA^43^ (Supplementary Fig. 2e).

To examine whether Xi-associated H3K27me3 was also redistributed in human nuclei undergoing reprogramming we performed immunofluorescence analyses. H3K27me3 labelled the Barr body in >80% of female hF, but this association was progressively lost in heterokaryons formed with mESCs (Fig. 2d,e). Three days after fusion most human nuclei (60%) lacked both *XIST* and H3K27me3 at the presumptive Xi. These results indicate that extensive remodelling of the human Xi occurs early in pluripotent reprogramming, ahead of hybrid cell formation and the first cell division.

### *ATRX* and *HUWE1* are reactivated early during reprogramming

**Figure 3:**
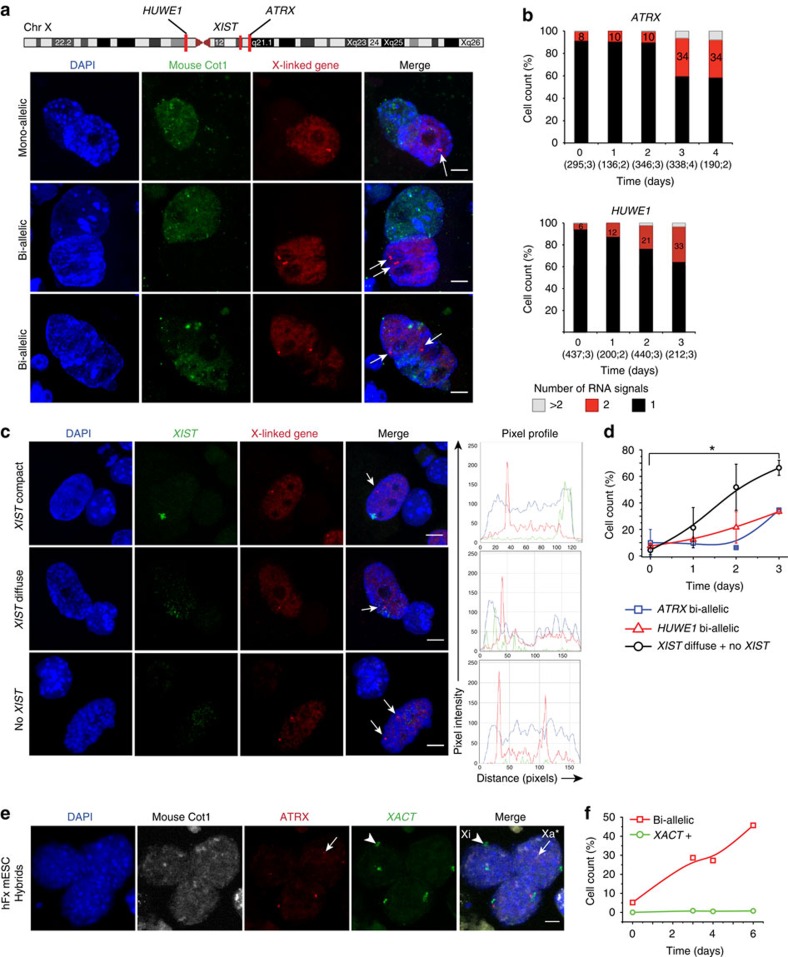
Bi-allelic expression of human X-linked genes is associated with *XIST* de-localization but not with *XACT* re-expression. (**a**) Confocal images show RNA-FISH for human *ATRX* or *HUWE1* nascent transcripts (red, arrowed) and mouse-specific Cot-1 repeats (green) in hFxmESC heterokaryons (top and middle panels) and hybrids (bottom panels). Scale bar, 5 μm. (**b**) Histogram plots represent the percentage of hFxmESC heterokaryons containing 1, 2 or >2 RNA signals. *ATRX* and *HUWE1* percentages at day 3/4 are significantly different from day 0 (*P*≤0.05; two-sided *t*-test). Total number of cells and number of replicate experiments are shown in parenthesis. Plots represent only heterokaryons; similar percentages of hybrids with two RNA signals were detected at day 4 (22% for *ATRX*; *n*=148). (**c**) Confocal images showing simultaneous detection of *XIST* RNA and nascent X-linked gene transcripts (that is, *ATRX* or *HUWE1*) in hFxmESC heterokaryons with distinct *XIST* distribution patterns. Profiles show the pixel intensity for DAPI (blue), *ATRX*/*HUWE1* transcripts (red) and *XIST* RNA (green) across the human nucleus; image scale=10,422 pixels μm^−1^. Scale bar, 5 μm. (**d**) Quantification of the patterns in (**c**). Cells that no longer have a compact *XIST* signal (that is, *XIST* diffuse+no signal) were scored together. Graphs represent the average of at least two independent experiments±s.e.m. and B-spline fitting (lines). (*) indicate significant changes (*P*≤0.05, two-sided *t*-test). Total number of cells scored at 0, 1, 2 and 3 days after fusion are 201, 168, 119 and 94 for *HUWE1* and 48, 99, 55 and 44 for *ATRX*, respectively. All scored cells were heterokaryons. (**e**) Confocal image showing three representative post-mitotic hybrids in which *XACT* RNA and nascent *ATRX* transcripts are simultaneously detected. *XACT* coats either one or two X chromosomes but none of the observed *XACT* patterns correlates with bi-allelic *ATRX* expression. Xa* indicates a putative reactivated X chromosome that transcribes *ATRX* but lacks *XACT*. Scale bar, 5μm. (**f**) Quantification of *XACT* positive and *ATRX* bi-allelic cells in (hFxmESC) heterokaryons and hybrids at successive days after fusion. Hybrids represented 30, 56 and 49% of total at day 3, 4 and 6, respectively. See also Supplementary Fig. 3.

Previous studies have indicated that loss of *Xist*/*XIST* does not lead to re-expression of X-linked genes in somatic cells^9^,^10^. To determine the transcriptional state of the human X chromosomes during reprogramming, we carried out RNA-FISH for two X-linked genes (*ATRX* and *HUWE1)* before and after the fusion of hF with mESC (Fig. 3). The majority of fibroblasts had a single RNA signal for *ATRX* and *HUWE1*, consistent with mono-allelic expression of both genes. We observed a progressive increase in the percentage of human nuclei with two discrete RNA signals in heterokaryons and hybrid cells following fusion (Fig. 3a,b), where co-labelling with mouse Cot-1 probe was used to discriminate human and mouse nuclei (as described in the online Methods). Sequential RNA/DNA-FISH confirmed that the detection of two RNA signals per nucleus (at more than 1μm distance) represents bi-allelic expression of X-linked genes (>20% at day 3), rather than locus replication (Supplementary Fig. 3a). We observed a similar proportion of heterokaryons showing two *HUWE1* or *ATRX* signals (approximately a third, Fig. 3b) and simultaneous RNA-FISH labelling of both loci (Supplementary Fig. 3b,c) confirmed that most cells showing bi-allelic expression of *ATRX* also expressed *HUWE1* bi-allelically, and *vice-versa* (Supplementary Fig. 3b,c). As expected, control hFxmB fused cells showed no evidence of bi-allelic expression of either of the genes (Supplementary Fig. 3c). These data showed that cell fusion-mediated pluripotent reprogramming of human fibroblasts could induce a switch from mono-allelic to bi-allelic expression of *ATRX* and *HUWE1*. To investigate whether *XIST* delocalization was likely to be a prerequisite for reprogramming-induced XCR we performed simultaneous RNA-FISH analysis for both *XIST* and *ATRX* (or *HUWE1*) in hFxmESC heterokaryons. This revealed that bi-allelic expression of *ATRX* or *HUWE1* was not significantly detected in cells retaining a ‘compact' *XIST* distribution, but was seen among cells in which *XIST* signals were diffuse or lost (Supplementary Fig. 3d) and that loss of Xi-associated *XIST* preceded the bi-allelic expression of selected Xi-genes in heterokaryons (Fig. 3d, 70% *XIST* diffuse or absent and 30% bi-allelic *ATRX*/*HUWE1*). These data are consistent with the delocalization of *XIST* from the Xi being required but insufficient for Xi gene reactivation.

The human long non-coding *XACT* RNA coats the Xa in human pluripotent cells^44^ (illustrated in Supplementary Fig. 3e) and has been implicated in erosion-mediated XCR in hESCs^27^. We, therefore, examined *XACT* expression during cell fusion-based reprogramming using RNA-FISH. *XACT* RNA was detected in a minor proportion (<1%, Supplementary Table 1) of hFxmESC fused cells from day 3 onwards and coated either one or both human X chromosomes. Importantly, *XACT* signals were exclusively seen in human hybrid cells (that is, were not detected before mitosis) and were not predictive of bi-allelic *ATRX* expression (illustrated in Fig. 3e,f). These data suggest that *XACT* induction and Xi reactivation are temporally uncoupled in this reprogramming scenario.

### Partial reactivation of the human Xi upon reprogramming

**Figure 4:**
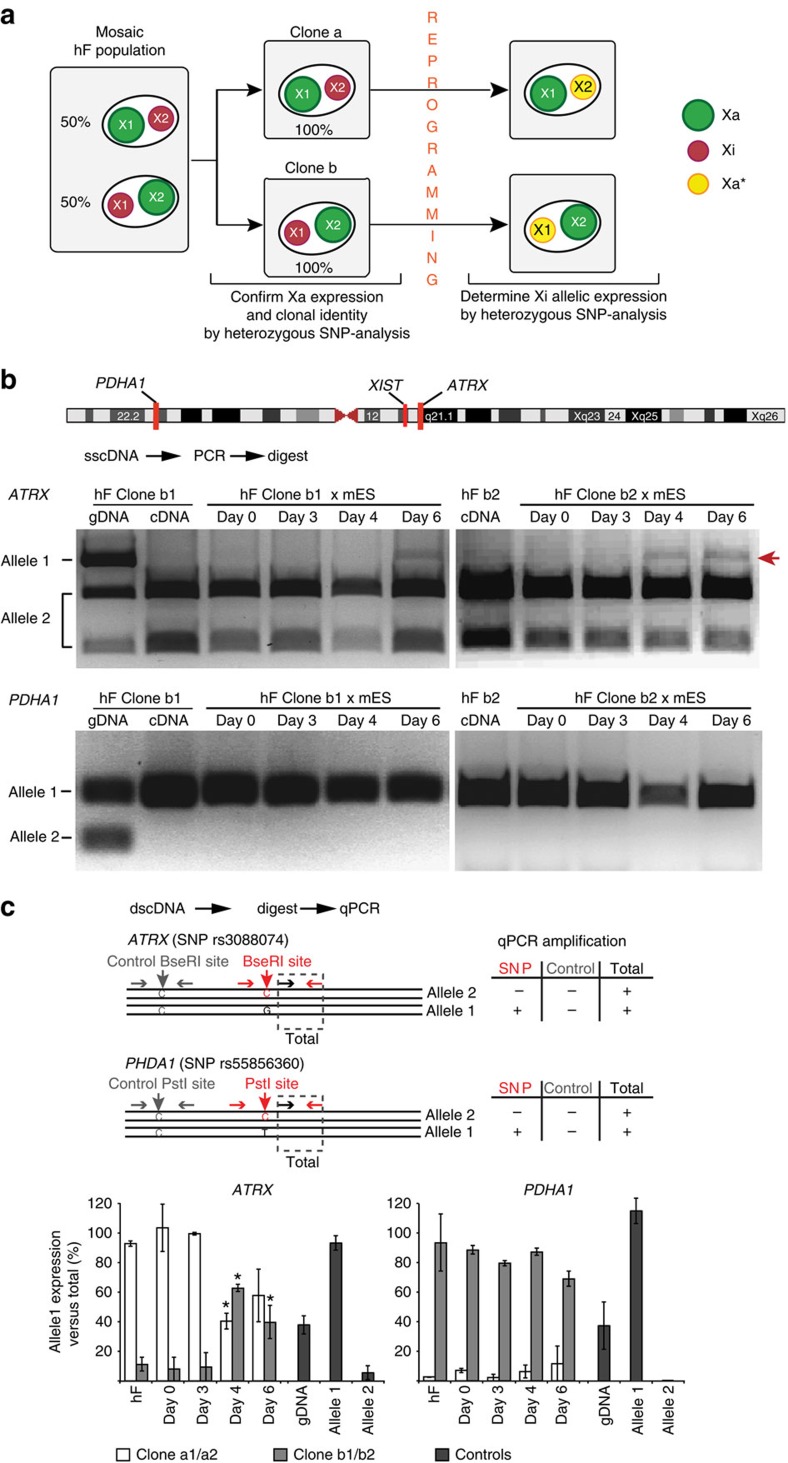
Allele-specific expression analysis of *ATRX* and *PDHA1* during cell fusion-mediated reprogramming reveals partial Xi reactivation. (**a**) Schematic representation of the experiments. Mosaic female hF (NHDF17914) that carry heterozygous SNPs within *ATRX* and *PDHA1* alleles on different X chromosomes (X^1^ and X^2^) were cloned by limiting dilution to obtain clones (type a or b) homogeneously expressing either one or the other allele (Xa^1^Xi^2^ or Xi^1^Xa^2^). SNP-specific expression analysis was then used to investigate reactivation of Xi-alleles upon hFxmESC fusion. (**b**,**c**) Two different experimental approaches showed reactivation of *ATRX* - but not *PDHA1* - upon mESC-fusion of different hF clones. In the first approach (**b**), *ATRX* and *PDHA1* alleles were analysed by enzymatic digestion of PCR products amplified from single-strand complementary DNA (sscDNA). Alleles on the two different X chromosomes generated restriction fragments of distinct lengths and were discriminated by agarose gel separation (restriction length polymorphisms analysis)^53^. Red arrow in the top panels highlights the *ATRX* allele that was reactivated upon hFxmESC fusions. In the second approach (**c**), restriction digestion was directly performed on double-strand cDNA (dscDNA) without prior amplification and a quantitative PCR strategy was used for determining expression of digestion-sensitive SNP and control sites along *ATRX* and *PDHA1* genes (top schematic and Methods). Histogram plots represent the percentage of allele 1 expression before and after fusion of human fibroblast clones. Data are shown as mean±s.e.m. of two independent experiments with a1/a2 (white bars) and b1/b2 (grey bars) clones. Genomic DNA from hF clones and plasmids with sub-cloned SNP sites for allele 1 or allele 2 were used as controls (black bars). (*) indicate values that significantly differ from hF/day 0 (*P*≤0.05, two-sided *t*-test). See also Supplementary Fig. 4.

To examine reactivation of Xi-specific alleles at the molecular level, we used single-nucleotide polymorphisms (SNPs) to distinguish transcripts originating from each of the two X chromosomes. To do this a human fibroblast cell line carrying heterozygous SNPs in *ATRX* and *PHDA1* genes was used to derive clonal populations where cells expressed the same X chromosome (X^1^ or X^2^; illustrated in the schematic representation shown in Fig. 4a). Both alleles of *ATRX* and *PDHA1* were expressed in the original human fibroblast line (hF complementary DNA), whereas only one of the two alleles was detected (X^1^a or X^2^a) in single-cell-derived clones (clone a1 and clone b1; Supplementary Fig. 4a,b), consistent with mosaic expression of X-linked genes and clonal epigenetic inheritance. To assess the reactivation of Xi-alleles (X^2^i or X^1^i), we reprogrammed these clones by fusing them with male mESCs (Supplementary Fig. 4c) and routinely detected the re-expression of the previously silent *ATRX* allele using two parallel approaches (Fig. 4b,c; described in the legends and methods), as well as by RNA-FISH analysis (Supplementary Fig. 4d). Although these data were consistent with previous analysis showing that bi-allelic expression of *ATRX* was induced upon reprogramming, another X-linked locus *PDHA1* appeared to be refractory to re-expression (Fig. 4b,c). This contrast in behaviour suggested that reprogramming-mediated reactivation might not be uniform along the Xi. Reactivation of *ATRX* but not *PDHA1* was consistently seen in fusions between different hF clones and mESCs (Fig. 4b,c) but not in control fusions with mouse somatic cells (Supplementary Fig. 4e). Importantly *XIST* transcript levels remained constant (Supplementary Fig. 4f), suggesting that *XIST* expression was not silenced in human cell fusion-mediated reprogramming.

**Figure 5:**
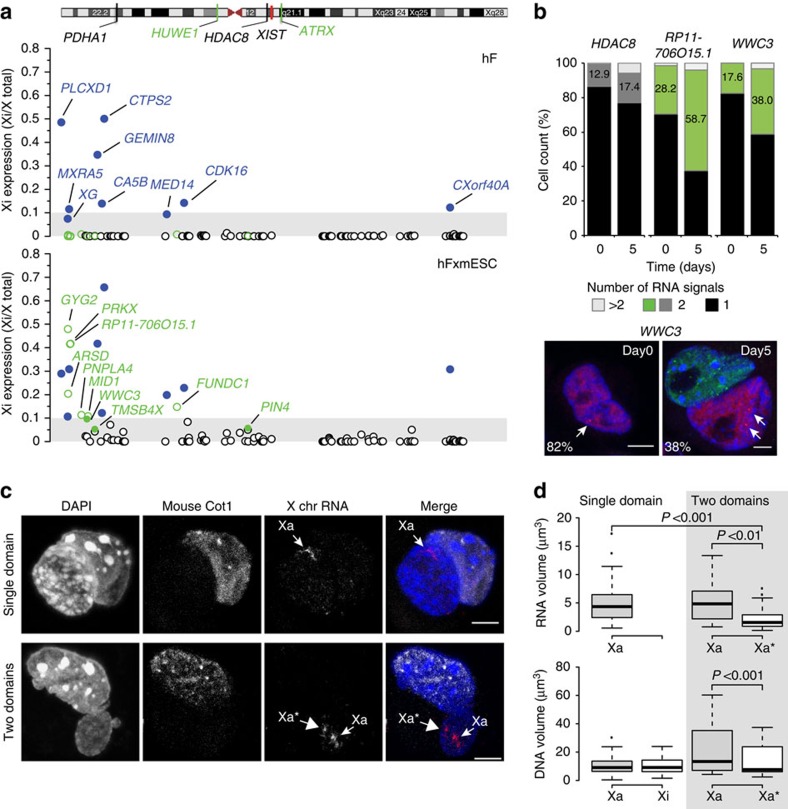
The human Xi is partially reactivated upon cell fusion-mediated reprogramming. (**a**) Expression of human Xi transcripts examined by allele-specific RNA-seq in female human fibroblasts (clone b1) before (day 0) and after (day 5/6) mESC-fusion. Data points show Xi expression of 113 genes along the X chromosome (*x* axis) where genes that escape XCI before reprogramming (blue closed circles),, or that are sensitive to reactivation following fusion (day 5 green open circles; day 6 green closed circles) are highlighted. Xi expression is represented as the ratio of reads overlapping the minor (Xi) allele versus the total of both alleles (Xi+Xa) and result from two biological replicates. Genes were classified accordingly to Xi expression ratio and significance^62^, as detailed in the Methods. (**b**) RNA-FISH analyses of candidate reactivation-refractory (*HDAC8*) and reactivation-sensitive (*RP11-706O15.1*, *WWC3*) genes in human fibroblasts before (day 0) and after mESC-fusion (day 5). Histogram plots show the percentage of human nuclei (heterokaryon/hybrid cells; *n*= 44/83, 33/74, 48/81 for *HDAC8*, *RP11-706O15.1* and *WWC3*, respectively) with one, two, or >2 punctate RNA signals. Representative confocal images showing mono-allelic expression of *WWC3* (red) in human nuclei before (day 0) and bi-allelic expression after (day 5) mESC-fusion (arrowed) where mouse nuclei were discriminated using mCotI probe (green). Arrows indicate transcribed loci (that is, punctate RNA signals). Scale bar, 5 μm. (**c**) Confocal images of hFxmESC heterokaryons where either one (top) or two (lower) transcribed X chromosomes (arrowed) were evident. Mouse Cot1 probe identifies mouse (ESC-derived) nuclei. X chr RNA paints were pre-treated with human Cot1 DNA to diminish the detection of repeat-rich elements^63^ within the transcribed X chromosome domains. Xa* marks the smaller (and Xa the larger) domain. Scale bar, 5 μm. (**d**) Box plots showing the volume of the RNA (top) and DNA (bottom) X domains detected by sequential RNA/DNA FISH in hFxmESC expressing one (single domain) or two (two domains) X chromosomes (shown in Supplementary Fig. 5b). Significant volume differences are highlighted (*P* values) and were estimated by Wilcoxon signed-rank test or Mann–Whitney test, for the comparison of Xa/Xa* and Xa/Xi, respectively. Data represent at least 50 cells in each graph. See also Supplementary Fig. 5.

To evaluate the extent of XCR we performed RNA-sequencing (RNA-seq) to determine allele-specific expression from the Xa and Xi of hF (clone b1) before and 5–6 days after mESC-fusion (Fig. 5a). Specifically, we analysed 113 human X-linked genes in which >20 SNP-overlapping reads were detected (Supplementary Data 1a). Among these, several genes showed evidence of XCI-escape (nine candidates shown in blue, Fig. 5a) being significantly expressed from the Xi by the parental clone ahead of fusion (day 0). Expression of several other Xi genes was selectively seen in samples after fusion (*GYG2*, *PRKX*, *RP11-706O15.1*, *ARSD*, *PNPLA4*, *MID1*, *FUNDC1*, *WWC3*, *TMSB4X*, *PIN4* shown in green), indicating reactivation-sensitive candidates, whereas other genes remained refractory to reactivation. To verify the status of candidates identified as being sensitive or refractory to Xi-reactivation by RNA-seq, we performed RNA-FISH to examine re-expression at the single cell level (Fig. 5b). In the case of controls, such as *HDAC8*, robust mono-allelic expression was observed before and after reprogramming, confirming that the Xi-allele of this gene was not reactivated. In contrast, bi-allelic expression of candidates such as *WWC3* and *RP11-706O15.1* was evident only after mESC-fusion (Fig. 5b), confirming sensitivity to XCR. Importantly, bi-allelic expression of such genes was evident within heterokaryons (as illustrated for *WWC3* in Fig. 5b) consistent with Xi reactivation occurring before the first cell division.

To further examine the extent of Xi-reactivation induced by reprogramming we performed RNA-FISH by using a mixture of commercially available probes that span the human X chromosome (X paint) but lack repetitive CotI elements^27^ (see methods). This allowed 'domains' of gene expression to be evaluated at the single cell level in hF before and after mESC-fusion (Fig. 5c and Supplementary Fig. 5a). A single transcriptional domain (Xa) was detected in individual fibroblast nuclei ahead of fusion, while two domains (Xa and Xa*) were evident in 24–38% of heterokaryons and hybrids examined at days 4–6 (Fig. 5c, Supplementary Fig. 5a,b). In these nuclei, we noted that one transcriptional domain (Xa*) was reproducibly smaller than the other (Xa), an observation that was confirmed by detailed volumetric assessment (Fig. 5d). To understand the basis for this difference, we examined hFxmESC in which either one (single domain) or two X transcriptional domains were detected (by RNA-FISH), using sequential X-DNA FISH to estimate the underlying chromosome volumes (Supplementary Fig. 5c). This analysis showed that although the reactivated Xa* chromosome occupied less volume than Xa in hFxmESC cells expressing two X domains, the Xi and the Xa* chromosome volumes were remarkably similar (DNA volumes in Fig. 5d), indicating minimal chromosomal de-condensation. These data suggest that pluripotent reprogramming had induced a partial reactivation of the Xi, consistent with RNA-seq analysis.

## Discussion

In this study, we have generated transient heterokaryons to examine the earliest events in human Xi-reactivation induced by pluripotent reprogramming. We have shown that upon fusion with mouse ESCs, female human fibroblasts re-express pluripotency genes and undergo a series of epigenetic changes that culminate in the re-expression of selected X-linked genes. Specifically reprogramming induced the loss of Barr body-associated histone H3K27me3 and delocalization of *XIST* from the human Xi, despite detection of *XIST* transcripts. These changes in pre-mitotic human chromatin appeared to be required for the subsequent re-expression of *ATRX* and *HUWE1*, but were insufficient to ensure the reactivation of many other X-linked genes including *PHDA1*. Previous reports based on studies of stable human–rodent hybrid clones had shown that *XIST* could delocalize from the Xi in the absence of pluripotent conversion^3^,^34^. However, we show here that *XIST* remained focused at the human Xi in control heterokaryons generated with somatic mouse B cells up until these cells enter mitosis. This observation, together with recent reports showing that *XIST* function relies heavily on a spectrum of different human RNA-binding proteins^45^,^46^,^47^, suggests that the reported delocalization of *XIST* in somatic cell hybrids probably reflects the paucity of human chromosomes retained by these cells rather than intrinsic species incompatibilities. In pre-mitotic heterokaryons, we found that *XIST* delocalization strongly correlated to pluripotency and preceded the bi-allelic expression of *ATRX* and *HUWE1* genes. Expression of human-specific *XACT* RNA was, in contrast, a much later temporal event that was only detected in a minority of hybrid cells after mitosis. This suggests that *XIST* and *XACT* have distinct roles in initiating and in maintaining Xi silencing and Xa activation, respectively. Although the role of *XACT* in XCR will require further study, the temporal separation of *XIST* removal and *XACT* detection shown here makes it unlikely that these two RNA species act as direct competitors in this reprogramming scenario. In human pluripotent cells, it has been suggested that *XIST* is antagonized by *XACT* binding along the X chromosome^27^. This hypothesis was based on the observation that *XACT* coats the Xa in ‘primed' hESCs (where *XIST* coats the Xi), and it localizes to the Xi ahead of *XIST* loss during the erosion of gene inactivation^27^. Here we show that *XIST* delocalization and partial Xi gene reactivation can occur in the absence of *XACT*. This could be because *XACT* expression requires the transition to a fully reprogrammed state, or it has a role in stabilizing XCR, or simply because it is not efficiently induced in an inter-species context.

The delocalization of *XIST* and loss of Barr Body-associated H3K27me3 reported here is consistent with previous *in vivo* studies showing that re-expression of *Oct4* in developing primordial germ cells is accompanied by loss of *Xist* and H3K27me3 (refs 48, 49). However, in contrast to what has been reported for hiPSCs 26, *XIST* expression was not silenced during cell fusion-mediated pluripotent reprogramming; rather, *XIST* displayed a change in nuclear localization. This redistribution could represent reduced binding of *XIST* to the Xi, or alternatively be the result of global de-condensation of the Xi in response to reprogramming and the influx of factors such as mouse-derived Oct4 (ref. 50). As we did not see evidence of any significant increase in the volume of the Xi during the first few days after cell fusion, our data support the former view that *XIST* dissociates from the Xi early during pluripotent reprogramming. This observation is consistent with studies in human blastocysts where, before the onset of XCI, *XIST* is reported to be present but is not tightly associated with the Xi^51^. Although our studies showed that *XIST* delocalization was insufficient to ensure global XCR, cells that re-expressed *ATRX* and *HUWE*1 lacked *XIST* or had a diffuse *XIST* distribution. This suggests that removal of *XIST* from the Xi is necessary but not sufficient for reprogramming-induced XCR. In the mouse, gene silencing relies on *Xist* for a brief period of time at the beginning of mouse ESC differentiation^52^. It is conceivable that reprogramming of somatic cells back to pluripotency might require a similar ‘window of opportunity' or transient state in which the removal of *XIST* from the Xi is required to reset gene activity.

Fusion of human somatic cells with mESCs triggers an influx of the mouse pluripotency factors into the somatic nucleus^37^. The subsequent re-expression of human pluripotency genes (*OCT4*, *NANOG*, *CRIPTO* and *REX1*) and later of *XACT-*RNA demonstrates that a human-specific pluripotency network is established following cell fusion and implies a certain degree of conservation between human and mouse in the mechanisms that lead to epigenetic reprogramming and XCR. Previous studies in iPSCs have shown that reactivation of the human Xi during pluripotent reprogramming is not achieved with standard human ESC medium^24^,^53^, but instead requires leukaemia inhibitory factor^54^ or GSK3/MEK inhibitors, together with constitutive overexpression of pluripotency factors^20^,^55^. Reprogramming using inter-species heterokaryons differs from iPSC-based reprogramming in several important respects and also offers some experimental advantages. For example, it allows access to events that occur before mitosis and cell division that are much less likely to depend on culture conditions. Using this approach, we reaffirm that XCI is partially reversed by pluripotent reprogramming of human somatic cells and describe an ordered sequence of epigenetic changes that occur ahead of the first cell division (Supplementary Fig. 6). We observed rapid and dynamic changes in histone H3K27me3 and *XIST* localization to the Xi that preceded bi-allelic expression of a small subset of X-linked genes. These data suggest that changes in chromatin organization occur ahead of the de-repression of selected X-linked genes. By examining heterokaryons and hybrid cells at later time points we confirmed that reactivation of the human Xi remains partial, with only 10% of the examined candidate genes switching to bi-allelic expression upon conversion towards pluripotency. Although we do not currently know the basis for the selectivity in genes showing reactivation this does not correlate with obvious features such as DNA methylation or proximity to CpG islands (personal communication, Bagci H.). Future studies will be required to identify factors and chromatin remodelling events that enable complete chromosome-wide Xi reactivation in human reprogrammed cells, as such knowledge is important to model X-linked diseases and for generating human iPSCs for regenerative medicine^56^.

## Methods

### Cell culture and fusion

Human fibroblast IMR90 (Coriell) and NHDF17914 (Lonza) were maintained in DMEM medium supplemented with 10% FCS, 1% non-essential amino acids, 2 mM L-glutamine and antibiotics (10 μg ml^−1^ penicillin and streptomycin). Primary hF cell lines were immortalized with pBABE-hTERT-blast and subsequently transduced with HP1αGFP-puroR retroviral construct (IMR90-hTERT-HP1αGFP-puroR). Single-cell clones were derived by limiting dilution in 3% O_2_ and clonal identity was confirmed by restriction-fragment length polymorphism analysis and Sanger sequencing of heterozygous SNP loci (Supplementary Fig. 4a,b) showing the homogenous expression of only one of the two X-linked genomic alleles (that is, a single X chromosome is expressed in each clone). Abelson-transformed mB and mESC (established in our lab) were cultured as previously described^37^. E14Tg2a *Hprt*^*−/*^mESC (gift from Niall Dillon) were transfected with pCAG-puro^57^ or pCAG-H2BmCherry-IRES-puro and stable clones were derived in 1.5 μg ml^−1^ puromycin. Human ESCs (H1 and H9 lines; gift from Wei Cui and Molly Stevens, respectively) were cultured in mTesR1 on Matrigel coated-dishes. For fusion experiments, cells were mixed in 1:1 ratio and treated in suspension with poly-ethylene-glycol (PEG 1500, Roche), as previously described^37^. Heterokaryons and hybrids were cultured in mouse ESC medium supplemented with mouse leukaemia inhibitory factor and selected after 12 h by adding puromycin (1.5 μg ml^−1^) and HAT (20 μM hypoxantine, 0.08 mM aminopterin, and 3.2 mM thymidine; Sigma), or puromycin (1.5 μg ml^−1^) and blasticidin (2 μg ml^−1^) for hFxmESC and hFxmB, respectively. Cells obtained upon fusion of GFP-positive hF with mCherry-positive mESCs were sorted twice at 24 and 48 h after fusion by FACSAriaIII (BD). All the cell lines used in this study have been regularly tested for mycoplasma contamination and resulted negative.

### Immunofluorescence and live-cell imaging

For immuno-staining, cells were grown on gelatin-coated coverslips, fixed with 2% paraformaldehyde for 15 min at room temperature and then permeabilized by 0.5% Triton-X-100 for 2 min and 0.1% Triton-X-100 for 30 min. Incubation in blocking buffer (2.5% bovine serum albumin, 0.05% Tween-20, 10% goat serum in 1 × PBS) was performed for 30 minutes before labelling with primary H3K27me3 antibody (1:500, Upstate 05-581 or Diagenode CS-069-100) for 1 h 30 min at room temperature. Cells were then washed by 0.2% bovine serum albumin/0.05% Tween-20 in PBS and stained with secondary antibodies (1:400, goat anti-mouse or goat anti-rabbit IgG Alexa Fluor 488 conjugate, Molecular probes A-11029 and R37116) and fluorescently-labelled phalloidin (1:100, Alexa Fluor 568 or 647; Molecular probes A12380 and A22287, respectively). Cells were finally mounted with Vectashield medium containing DAPI (Vector labs) and imaged on a Leica SP5 II confocal microscope. Human and mouse nuclei were distinguished based on DAPI-staining and the presence of chromocenters in the mouse nuclei. Individual cells were delineated by phalloidin and inter-species heterokaryons were identified as cells containing two or more nuclei both of human and mouse origin, as illustrated in Supplementary Fig. 1c.

Cells obtained after fusion between HP1α-GFP hF with H2B-mCherry mESCs were attached on poly-L-lysine treated-coverslips immediately after sorting. In this case, hybrids obtained upon fusion of the human and mouse nuclei could be detected as cells with GFP+/Cherry+ single nuclei (Supplementary Fig. 1c,d). For time-lapse microscopy, sorted cells were plated on gelatinized dishes (35 mm μ-Dish, IBIDI) and imaged every 3 min for 18 h. Images were acquired at Leica SP5 II confocal with × 63/1.4NA HCX PL APO lambda blue objective and processed by ImageJ^58^.

### RNA and DNA-FISH

RNA and DNA-FISH were performed as previously described^7^. Probes used in this study were obtained by nick translation (Abbott Molecular) of plasmids, bacterial artificial chromosomes or mouse Cot-1 DNA (Invitrogen) in the presence of fluorophore-conjugated dUTPs (ENZO). Bacterial artificial chromosomes used for the synthesis of *ATRX*, *HUWE1*, *HDAC8*, *RP11-706O15.1* and *WWC3* probes were RP11-42M11, RP11-155O24, RP11-1021B19, WI2-1543K8 (CHORI), and CTD-2277I2 (Invitrogen), respectively. *XIST* probes were synthesized from G1A plasmid (gift from J.B. Lawrence) containing 10 kb from the fourth intron to the 3′-end of the gene, and from a plasmid containing *XIST* exon1.

RNA-FISH for detecting transcribed domains on the human X chromosome was performed by using human X chromosome paint (Cambio), as previously detailed^27^. Briefly, 5 μl of concentrated X paint was mixed with 5 μg of human Cot1 DNA (Life Technologies), denatured for 7 min at 75 °C and pre-annealed for 30 min at 37 °C in order to deplete the probe of Cot1 repeats before hybridizing with cells overnight at 37 °C.

In order to identify heterokaryons and hybrids, slides were automatically acquired at low resolution with a × 20/0.7NA CS HC PL APO objective by using Leica SP5 confocal microscope and LAS AF Matrix Screener software. Images were then analysed by ImageJ to find the coordinates of heterokaryons that showed partially overlapping signals for mouse-specific Cot-1 repeats and human-specific X-linked gene probes. Selected positions were then acquired at higher resolution by × 63/1.4NA HCX PL APO lambda blue objective (*z*=0.2 μm). Images were processed and analysed by ImageJ. Volumetric analysis was performed by NEMO/3D-FISH plugin^59^.

### RNA extraction and RT–PCR

Total RNA extraction and SYBR-Green RT–qPCR were performed as previously described in ref. 37. Primers used in this study are reported in Supplementary Table 2. *XIST* allele-specific expression (Supplementary Fig. 4f) was analysed by Taqman PCR performed with SNP genotyping assay (rs1620574; assay ID C-7626536_10 Applied Biosystems) and Taqman Universal Master Mix No AmpEraseUNG (Applied Biosystems) according to the manufacturer instructions.

### SNP-restriction assays

Restriction-fragment length polymorphism analysis for *ATRX* and *PDHA1* was carried out as previously described^53^. Specificity of the primers for human transcripts was validated by performing RT–PCR on mESCs. For quantitative analysis of restriction digestion-sensitive SNPs (Fig. 4c), double-stranded complementary DNA was generated from 1 μg total RNA via SuperScript kit (Invitrogen), purified with Agencourt AMPure XP beads (Beckman Coulter) and digested by *Bse*RI or *Pst*I for *ATRX* and *PDHA1*, respectively. SYBRgreen PCR was performed upon digestion by using primers reported in Supplementary Table 1. Allele1 expression was quantified using primers spanning the restriction-sensitive SNP site (red arrows in Fig. 4c) and normalized versus the total expression of allele1 and 2 (amplified by the black and red primers in the dashed rectangle; Fig. 4c). Allelic ratios were further normalized for the digestion efficiency, which was estimated by quantifying the expression of a control restriction site within the same exon containing the SNP site (grey arrows).

### RNA-seq and bioinformatic analysis

RNA-seq libraries were prepared from 0.6 μg of total RNA (RNA integrity number (RIN) >7.5) by True-seq RNA sample prep v2 kit (Illumina) and were amplified by 13 PCR cycles. Paired-end 100 bp reads were generated using HiSeq2500 sequencer (Illumina). RNA-seq reads from two biological replicates of hF clone b1 before (day 0) and after (day 5) fusion were aligned independently to human (hg19, Ensembl gene version 72) and mouse reference genomes (mm9, Ensembl gene version 67) using Tophat v2.0.8 (ref. 60). The sequencing reads aligning to both human and mouse genomes were excluded and only human-specific reads were considered for allelic expression analysis. In order to estimate allelic expression, we used a set of 379 heterozygous SNPs previously identified across 183 genes by performing RNA-seq of two isogenic clones with opposite haplotype expression (for example, a1 and b1) and selecting genomic positions with a different base in the two clones. The number of SNP-overlapping reads for each allele was obtained using SAMtools mpileup^61^. At each SNP position the allele representing the majority of reads in hF before fusion (day 0) was considered as the major allele (Xa) and the other the minor allele (Xi). Xa- and Xi- alleles identified at day 0 were then used to compare allelic expression in samples after fusion. Allelic expression was calculated as the sum of reads overlapping the Xa or Xi alleles (identified by SNPs) for each gene, and is represented as ratio of Xi/(Xa+Xi). A gene was considered expressed from the Xi when its Xi ratio was higher than 10% or when it had a significant ‘probability of escaping XCI' (that is, being expressed from the Xi, *τ*0 ≤0.05) based on a previously described beta-binomial model^62^. Briefly, this allows the probability that a gene is expressed from the Xi to be estimated based on the total number of reads obtained from the Xi allele, the sum of the base quality scores for the Xi-SNPs within these reads, and the Xi/(Xi+Xa) ratio. RNA-sequencing data were fitted to a mixture of beta-binomial distribution in order to determine significant Xi expression and obtain the probability of escape (*τ*0). To increase confidence in annotation, only genes with minimum 20 SNP-overlapping reads at day 0 and at day 5 were considered (*n*=113, Supplementary Data 1).

Genes were classified into three groups according to allelic expression in hF clone b1: (i) genes that escaped XCI and showed significant Xi expression before (day 0) and after fusion (day 5) (Fig. 5a and Supplementary Data 1, blue), (ii) genes that showed significant Xi expression after fusion (day 5) but not before (day 0) and represent reactivation-sensitive candidates (shown in green, Fig. 5a and Supplementary Data 1) and (iii) genes that were refractory to Xi reactivation and showed no significant Xi expression at day 0 or after fusion (shown in black, Fig. 5a and Supplementary Data 1). Additional analyses of samples obtained slightly later after fusion were performed as outlined above and identified three additional reactivation-sensitive genes that had significant Xi expression at day 6 (but not at day 0): *WWC3*, *TMSB4X* and *PIN4* (Fig. 5a and Supplementary Data 1b).

### Data availability

RNA-seq data set has been submitted to gene expression omnibus (GEO), with accession number GSE77443.

## Additional information

**How to cite this article:** Cantone, I. *et al.* Ordered chromatin changes and human X chromosome reactivation by cell fusion-mediated pluripotent reprogramming. *Nat. Commun.* 7:12354 doi: 10.1038/ncomms12354 (2016).

## Supplementary Material

Supplementary Information Supplementary Figures 1-6 and Supplementary Tables 1-2
