# Supplementary material for: Ordered chromatin changes and human X chromosome reactivation by cell fusion-mediated pluripotent reprogramming
Source: Nat Commun. 2016 Aug 10;7:12354. doi: 10.1038/ncomms12354 (PMC4987517; doi:10.1038/ncomms12354)
Supplement: Supplementary Information — Supplementary Figures 1-6 and Supplementary Tables 1-2 [file ncomms12354-s1.pdf]

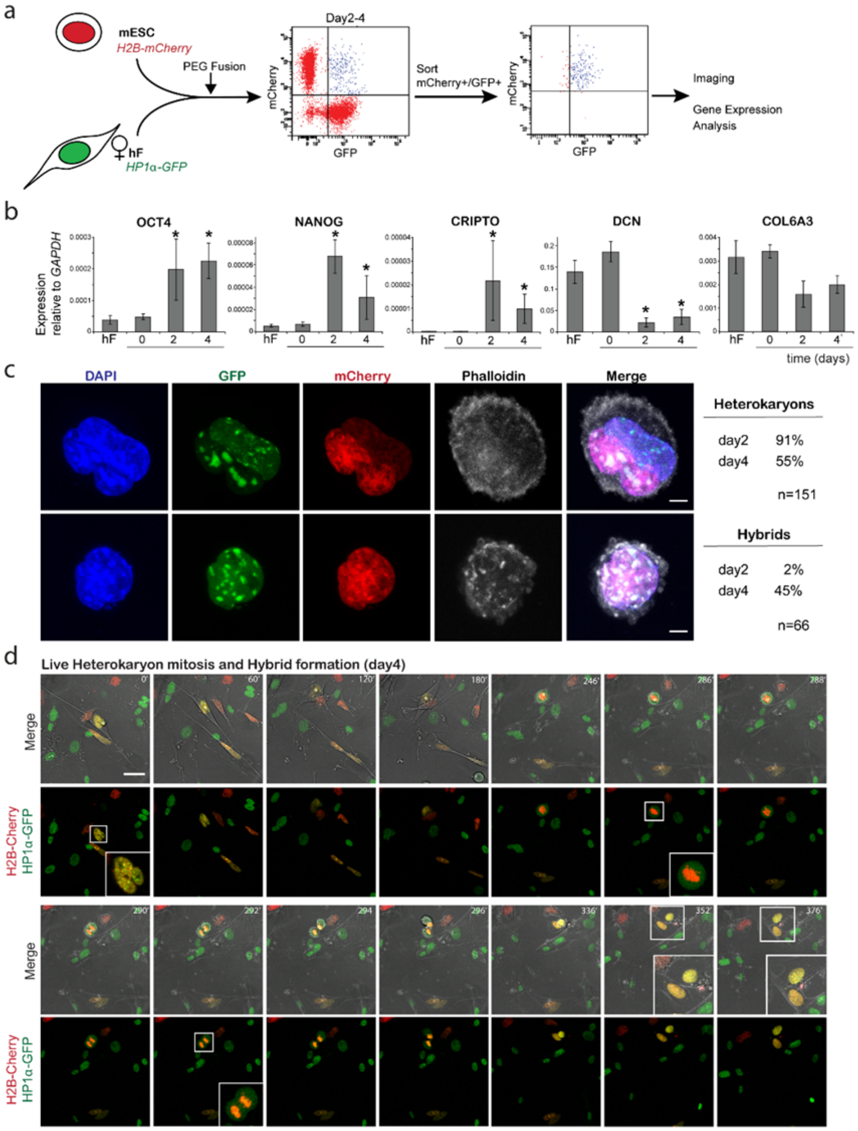

Supplementary Figure 1. Pluripotent reprogramming begins in transient hFxmESC heterokaryons before cell division. (**a**) Scheme of the approach used to evaluate the kinetics of cell fusion-mediated pluripotent reprogramming. Female human HP1α-GFP-fibroblasts (hF, IMR90hTERT) were fused with H2B-mCherry-mESCs (E14Tg2a) and double positive cells (GFP+/mCherry+) isolated by FACS and used in gene expression (b) and imaging (c) analysis. (**b**) Histogram plots showing the relative expression ($2^{-\Delta Ct}$) of human pluripotency genes (*OCT4*, *NANOG*, *CRIPTO*) and fibroblast-specific genes (*DCN*, *COL6A3*). Values are the mean of 2 to 4 experiments ±SEM and (*) indicate significant differences from hF ($p \leq 0.05$, t-test). (**c**) Confocal images of representative heterokaryon (top) and hybrid (bottom) cells. GFP+/mCherry+ cells were isolated by FACS at days 2 and 4, labelled with phalloidin and DAPI stain, and examined by microscopy to evaluate the kinetics of heterokaryon and hybrid formation (right). Hybrid cells were evident 3 to 4 days after cell-fusion. n=222 (day 2); n=66 (day 4). Scale bars =5μm. (**d**) Confocal time-lapse images show a representative heterokaryon undergoing nuclear fusion and mitosis at day 4. Merge images represent fluorescent channels combined with the bright field where cell membranes are visible. Cells were sorted only once before re-plating and imaging. Unfused hF (green) and mESC (red) provide controls. Scale bar =50μm.

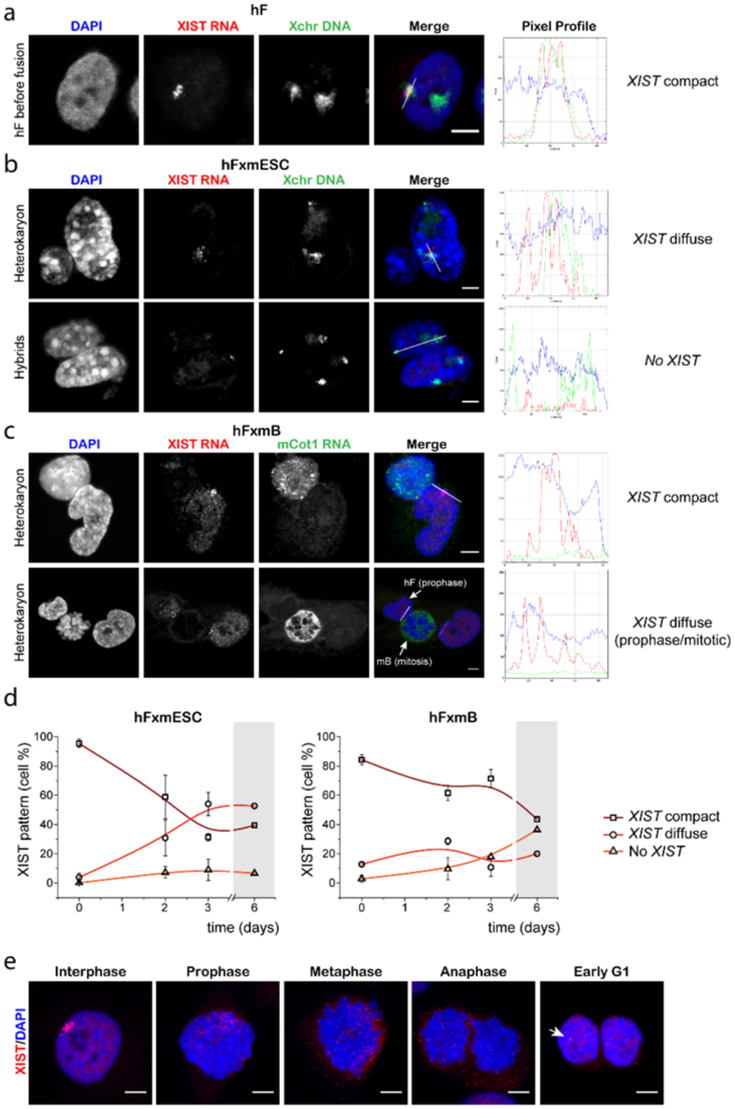

Supplementary Figure 2. *XIST* delocalisation is not induced upon fusion with mouse B cells. (**a**)-(**c)** Confocal images (left) and pixel profiles (right) of *XIST* RNA distribution in representative hF nuclei before fusion (**a**) and in hFxmESC heterokaryon and hybrid cells (**b**) where human X-chromosome territories were revealed using DNA probes (Xchr). Scale bars =5µm. (**c**) *XIST* distribution in heterokaryons formed between female hF and mB cells (hFxmB) was examined by RNA-FISH, where mouse Cot-1 probe was used to discriminate mouse nuclei. Compact *XIST* signals were detected in the majority of hFxmB cells (top panels). In cells entering mitosis *XIST* signal was either diffuse or absent (bottom panels) and these mitotic cells were abundant 6 days after hFxmB fusion (35% of cells with diffuse or no *XIST* signal quantified in **d**). Scale bars =5µm. (**d**) Quantification of heterokaryons with a compact (-O-), diffuse (-□-) or no (-△-) *XIST* signal in hFxmB (right) and hFxmESC (left; data from Figure 2b are shown for comparison). Total number of scored cells is 427, 170, 28 and 55 (hFxmB) and 790, 298, 196 and 180 (hFxmESC) at 0, 2, 3 and 6 days after fusion. Data are represented as mean ± SEM. Trendlines are fitted with B-spline function. (**e**) Confocal images showing representative examples of *XIST* RNA signal in human fibroblasts at different cell cycle stages. *XIST* RNA is diffuse in mitotic nuclei and re-localises in G1 phase (arrowed) as cells enter a new cell-cycle. Scale bars =5µm.

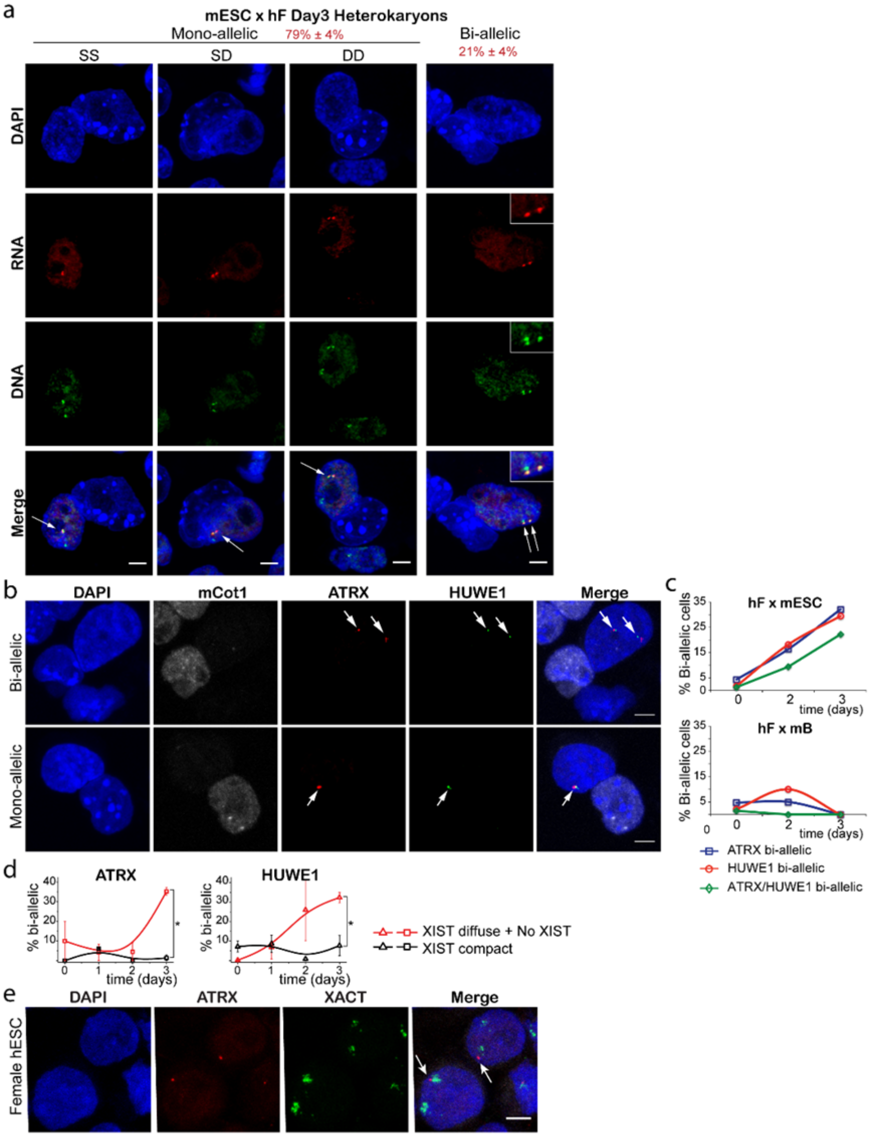

Supplementary Figure 3. Bi-allelic expression of human X-linked genes upon pluripotent reprogramming. (**a**) Confocal images showing sequential RNA (red) and DNA (green) FISH of *HUWE1* loci in day 3 hFxmESC heterokaryons, where doublet DNA signals represent replicated sister chromatids that are spatially separated (2 close pinpoints at less than 1µm distance) and 'singlets' are either un-replicated or spatially-unresolved replicated alleles. Mono-allelic expressing cells with two singlet (SS), one singlet and one doublet (SD) or two doublet (DD) DNA signals show RNA emanating from only one of the two alleles, whereas bi-allelic *HUWE1* expression (right) was easily recognised. Arrows indicate the expressed alleles in the human nuclei. Scale bars =5µm. (**b**) Confocal images of representative hFxmESC heterokaryons showing bi-allelic (top) or mono-allelic (bottom) expression of *ATRX* (red) and *HUWE1* (green) by simultaneous RNA-FISH. Arrows indicate the nascent RNA transcripts. Scale bars =5µm. (**c**) Quantification of bi-allelic cells for *ATRX* (blue squares), *HUWE1* (red circles) and both *ATRX/HUWE1* (green diamonds), in hFxmESC (top) and hFxmB (bottom) heterokaryons. Total number of cells is 230, 274 and 112 (hFxmESC) at 0, 2 and 3 days after fusion, and 274, 20, and 29 (hFxmB) and represents only heterokaryons. (**d**) Quantification of simultaneous RNA-FISH for *ATRX* (or *HUWE1*) and *XIST* in hFxmESC heterokaryons (representative images are shown in Figure 3c). Graphs show the percentage of bi-allelic expressing

cells with compact (black) or delocalised (i.e. diffuse + absent, red) *XIST* signals. Data represent the average of at least two independent experiments ± SEM and B-spline fitting (lines). (*) indicate significant changes ($p \leq 0.05$, t-test). (**e**) Confocal images showing simultaneous FISH detection of *XACT* RNA and *ATRX* nascent transcripts (arrowed) in human ESCs with partial erosion of XCI. Scale bars =5μm.

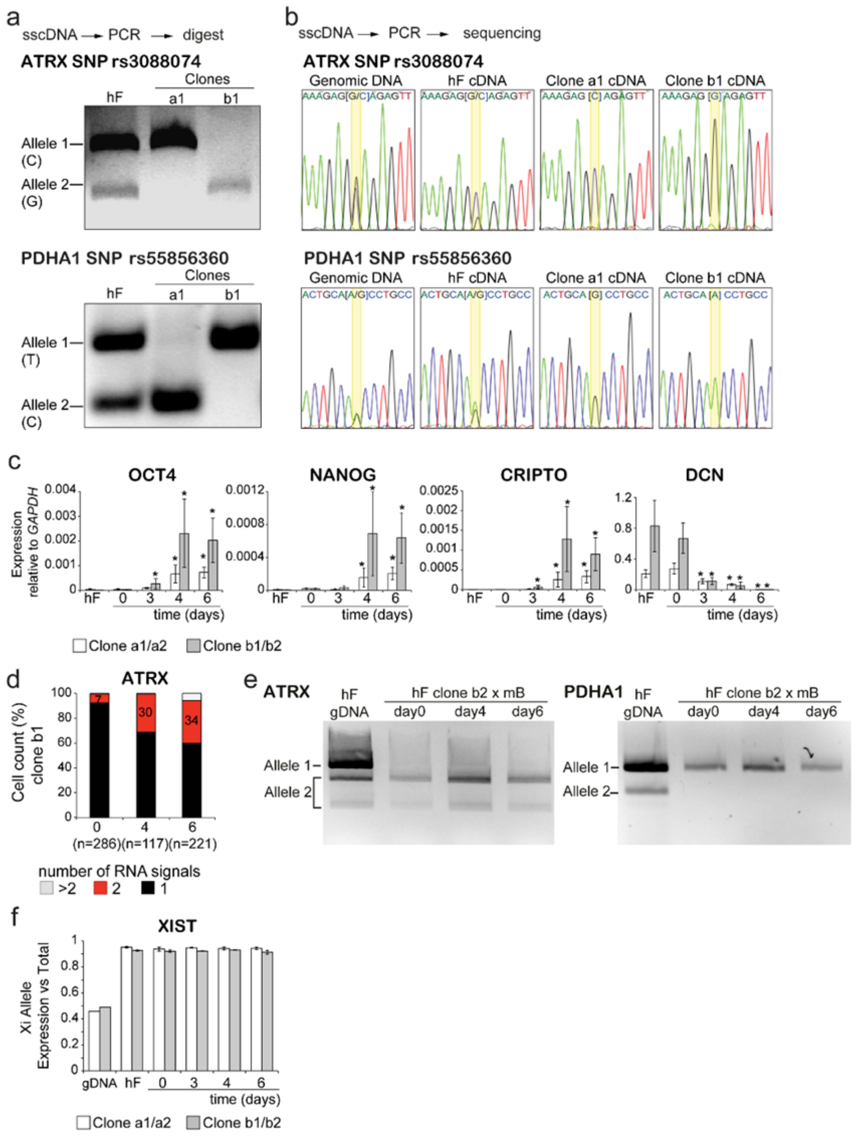

Supplementary Figure 4. Allele-specific expression analysis of *ATRX* and *PDHA1* in different human fibroblast clones. (**a**) RFLP expression analysis of SNP sites in *ATRX* (top panel) and *PDHA1* (bottom panel) gene. Restriction digestion of RT-PCR products generates fragments of distinct lengths from alleles on the two different X chromosomes. This shows that clones a1 and b1 express only one of the two alleles that are detected in the original mosaic hF population. (**b**) Sanger sequencing of RT-PCR products from clones a1 and b1 before mESC-fusion confirm homogeneous *ATRX*/*PDHA1* SNP expression in clonal fibroblasts (yellow highlighted peaks). Double peaks are visible in a representative example of genomic DNA showing the presence of both alleles in the genome of the clones. (**c**) Histogram plots showing the expression of human pluripotency genes (*OCT4*, *NANOG*, *CRIPTO*) and fibroblast-specific genes (*DCN*) in hF clones a1/a2 (white bars) and b1/b2 (grey bars) before and after mESC-fusion. Data represent the average of at least 3 independent experiments performed with different clones ± SEM. (*) indicate statically significant changes from hF ($p \leq 0.05$, t-test). (**d**) Quantification of heterokaryon and hybrid cells showing one, two or more than two transcribed *ATRX* loci by RNA-FISH labelling of clone b1 fibroblasts before and after fusion with mESC. Scores at day 4 and 6 represent 12% of heterokaryons. (**e**) RFLP analysis of *ATRX* and *PDHA1* expression at 0, 4 and 6 days upon fusion of hF clone b2 with mB cells and in the hF clone b2

genomic DNA (gDNA). (**f**) Histogram plots showing the allele-specific expression of *XIST* in hF clones before and at 0, 3, 4, and 6 days after mESC-fusion. Data are obtained by SNP-specific Taqman PCR and are represented as expression of the Xi allele versus total of both alleles (i.e. normalised relative fluorescence units). No Xa transcripts were detected suggesting constant mono-allelic expression of *XIST* from the Xi. Error bars indicate SEM of two independent experiment performed with different hF clones.

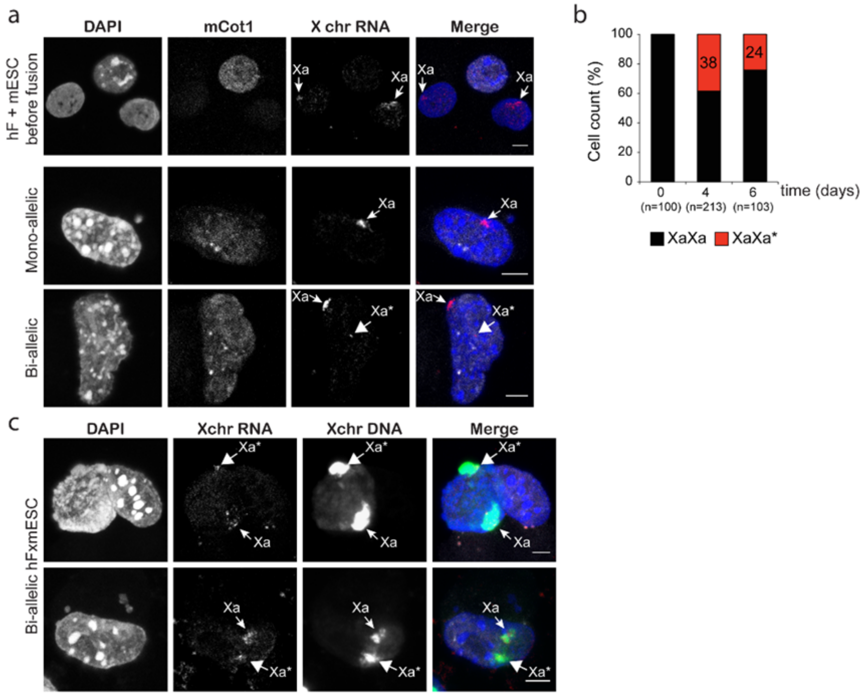

Supplementary Figure 5. RNA-FISH labelling of transcribed X chromosome territories confirm partial Xi reactivation during cell fusion-mediated reprogramming. (**a**) Confocal images showing simultaneous FISH detection of mouse Cot-1 (mCot1) RNA and transcribed X chromosome domains in hF and mESC before fusion (top panels) and in hFxmESC hybrids with one (middle panels) or two (bottom panels) transcribed X chromosomes. Scale bar = 5µm. (**b**) Histogram plots showing the percentage of hFxmESC heterokaryon and hybrid cells with one (black) or two (red) active X chromosomes during cell fusion-mediated reprogramming. Number of cells that were scored in two independent experiments is reported in parenthesis along the x axis. 24% and 12% of scored cells are heterokaryons at 4 and 6 days respectively. (**c**) Confocal images showing sequential RNA/DNA-FISH with whole X chromosome paint probes (Cambio) in a representative heterokaryon (top) and hybrid (bottom) cell. Xa* marks the X chromosome with the smaller transcribed X domain (Xchr RNA). Scale bar = 5µm.

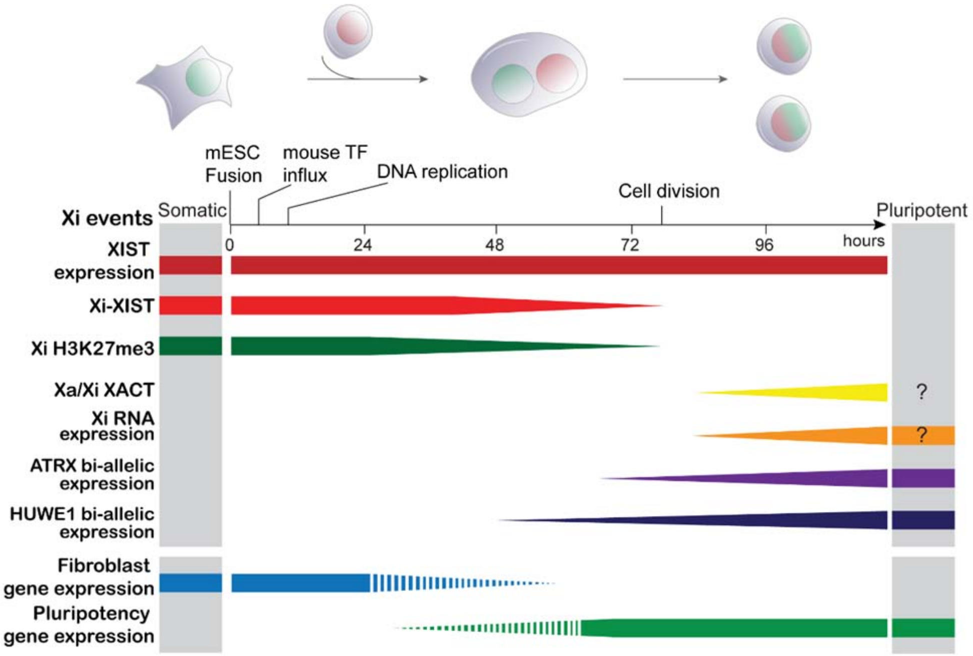

Supplementary Figure 6. Summary of temporal events induced at the human Xi by cell fusion-mediated pluripotent reprogramming.

Supplementary Table 1. Quantification of *XACT* positive and *ATRX* bi-allelic expressing cells [1]

	Days after fusion			
	**0**	**3**	**4**	**6**
**Bi-allelic *ATRX***	5%	29%	27%	46%
***XACT* +**	0%	0.9%	0.5%	0.8%
**n**	289	174	87	66

[1] Data represented in Figure 3f

Supplementary Table 2. Primer sequences

Primer	Sequence	Assay
GAPDH F	TCTGCTCCTCCTGTTCGACA	RT-qPCR
GAPDH R	AAAAGCAGCCCTGGTGACC	RT-qPCR
OCT4 F	TCGAGAACCGAGTGAGAGGC	RT-qPCR
OCT4 R	CACACTCGGACCACATCCTTC	RT-qPCR
NANOG F	CCAACATCCTGAACCTCAGCTAC	RT-qPCR
NANOG R	GCCTTCTGCGTCACACCATT	RT-qPCR
CRIPTO F	AGAAGTGTTCCCTGTGTAAATGCTG	RT-qPCR
CRIPTO R	CACGAGGTGCTCATCCATCA	RT-qPCR
REX1 F	GCGTACGCAAATTAAAGTCCAGA	RT-qPCR
REX1 R	TCAGATCCTAAACAGCTCGCAGAAT	RT-qPCR
DCN F	TCCTCAAGGTCTTCCTCCTTCCCTT	RT-qPCR
DCN R	GCGTGTTGGCCAGAGAGCCATT	RT-qPCR
COL6A3 F	TTTGCTCAGGGGTTCATATTTGG	RT-qPCR
COL6A3 R	AGCAGCCGCACCATTTTTGAC	RT-qPCR
XIST 1F	TTGCCCTACTAGCTCCTCGGAC	RT-qPCR
XIST 1R	TTCTCCAGATAGCTGGCAACC	RT-qPCR
XIST 2F	ACGCTGCATGTGTCCTTAGTAGTC	RT-qPCR
XIST 2R	ATTTGGAGCCTCTTATAGCTGTTTG	RT-qPCR
XIST 3F	ACTTGGATGGGTTGCCAGCTA	RT-qPCR
XIST 3R	GCCCCATCTCCACCTAGGGA	RT-qPCR
XIST 4F	TCTACTTGGATGGGTTGCCAGC	RT-qPCR
XIST 4R	TGCCCCATCTCCACCTAGGGA	RT-qPCR
ATRX F Allele2/Total	TCTGATGATGCTGAAAGAAAACA	RFLP qPCR assay[1]
ATRX R Total	CATCAGTGGAAGCACTTGCT	RFLP qPCR assay[1]
ATRX R Allele2	TCAGCAACTTTTCTAACTTCCAA	RFLP qPCR assay[(1)]
ATRX F Control	ATGGCAGCAGTGGAACTGA	RFLP qPCR assay[(1)]
ATRX R Control	CTTCCTGACAATCAGCACCT	RFLP qPCR assay[(1)]
PDHA1 F Total	AGAAGGGGGTGGGTCCAG	RFLP qPCR assay[(1)]
PDHA1 R Total/Allele1	GGAATGTGACAGAGAAGAGCT	RFLP qPCR assay[(1)]
PDHA1 F Allele1	GACTCGGGAACAAGAAGGCA	RFLP qPCR assay[(1)]
PDHA1 F Control	GCTCGTGTCCCAGCAGTAGT	RFLP qPCR assay[(1)]
PDHA1 R Control	GCCTTCTTGTTCCCGAGTCAT	RFLP qPCR assay[(1)]

[(1)] RFLP qPCR assay strategy is described in Figure 4c and online Methods.
